# Support Desired by Parents of Infants With Hearing Impairments Diagnosed Through Newborn Hearing Screening: A Questionnaire-Based Survey

**DOI:** 10.7759/cureus.75482

**Published:** 2024-12-10

**Authors:** Satomi Shojinaga, Haruo Yoshida, Yukihiko Kanda, Yoshihiko Kumai, Haruo Takahashi

**Affiliations:** 1 Department of Otolaryngology - Head and Neck Surgery, Nagasaki University Graduate School of Biomedical Sciences, Nagasaki, JPN; 2 Department of Otolaryngology - Head and Neck Surgery, National Hospital Organization Nagasaki Medical Center, Ōmura, JPN; 3 Department of Otolaryngology - Head and Neck Surgery, Kanda ENT Clinic, Nagasaki Bell Hearing Center, Nagasaki, JPN

**Keywords:** cochlear implantation, congenital hearing loss, early intervention, education, hearing aid, parental needs, quality of life, social support

## Abstract

Objective

We aimed to highlight problems faced by parents of infants diagnosed with hearing impairment upon newborn hearing screening (NHS) and to suggest how support might be improved.

Methods

We distributed a questionnaire to explore difficulties encountered by parents when seeking support, whether they were satisfied with the support, and their unmet needs. We enrolled 101 parents of infants with hearing impairments diagnosed upon NHS (hearing levels: 7.0-105.0 dB, mean: 51.5 dB, standard deviation: 27.7 dB). The results were compared by hearing level, current child age, and residential area.

Results

Only 46 (46%) parents were satisfied with the support received, and nine (27%) of those whose children exhibited normal hearing were moderately dissatisfied. Medical care was viewed as good, but administrative support was seen as poor; financial assistance was lacking, and regional disparities were apparent. The hearing levels of children whose parents were dissatisfied with total support were statistically significantly lower than those of children whose parents were satisfied (p = 0.0263). Since the quality of medical care has been improving year by year, it may be inferred that, as a result, parents of more recently born children are more satisfied with their medical support. Parents of children with poor hearing levels were more satisfied with educational support than others, perhaps because their children attended special schools.

Conclusions

There is a need to address issues perceived by parents of children with hearing impairments. The support available, and ways to access that support, must be clarified. Regional disparities in support policies and education must be eliminated, and support requires more human resources.

## Introduction

Newborn hearing screening (NHS) and the confirmation of hearing loss in infants mean little in the absence of appropriate, prompt, individualized, targeted, high-quality intervention [1]. Early intervention must include both hearing amplification (or other treatment) and family support. Although the Parent Satisfaction with Newborn Hearing Screening Programs Questionnaire (PSQ-NHSP) has been evaluated in many countries [2,3], it assesses only satisfaction with the NHS per se, not with follow-up support. Many studies have described the interventions, medical care, or other treatments for infants with NHS-diagnosed hearing loss, whereas few have evaluated parental support. The Joint Committee on Infant Hearing stated that evaluating parental satisfaction is important to assessments of NHS efficacy [4]. It is essential to explore how parents perceive the audiological management of infants with hearing loss [5-11]. Parental stress reduces the quality of the parent-child relationship and is associated with poor child developmental outcomes [11]. If parents of children with hearing loss are severely stressed, their children may experience poor social and emotional outcomes, behavioral problems, and low quality of life [12-15]. Thus, the fulfillment of parental support needs would reduce parental stress and improve parent-child relationships.

The purpose of this study is to clarify the level of satisfaction felt by parents of children suspected to have hearing loss in the NHS regarding each type of support and to suggest necessary responses and remedial measures to address them.

## Materials and methods

Study design and setting

This was a cross-sectional, questionnaire-based study. This study was conducted at Nagasaki University Hospital in Nagasaki, Japan.

Participants

All participants were parents of children born between January 2010 and December 2019 who were NHS-diagnosed with hearing loss. During this period, a total of 1,412 infants were referred to our institution, of whom 413 (29.2%) required follow-up at >1 year of age due to either hearing loss or inconclusive results on the hearing test. We retrieved the addresses of all 413 cases from our hospital database and mailed questionnaires to all parents in December 2020. During the two-month data collection period, 124 respondents agreed to participate in the survey (return rate: 30.0%). Of these, we excluded 23 (18.5%) parents with children for whom hearing data were lacking, or whose answers did not allow full statistical analysis. Thus, the parents of 101 (81.5%) children (48 girls and 53 boys) were included in this study. Parents who did not contact us within four weeks of the questionnaire mailout, and those who were not reached at the addresses in our database, were considered non-responders.

Questionnaire survey

We classified support for children with hearing loss as total, medical, educational, administrative, and familial support, and analyzed parental needs using a questionnaire. Medical care included medical examinations, explanations to parents, and regular follow-ups. Education covered special seating arrangements in schools and the use of hearing aids. Administration included subsidies for the purchase of hearing aids and allied systems. Familial support covered home treatment and transportation to and from the hospital.

A literature search did not yield a validated questionnaire that measured the satisfaction of parents of infants with NHS-diagnosed hearing loss. Therefore, we developed a questionnaire and conducted a pilot study in October 2020, including five mothers of children with normal hearing who worked at our University Hospital. Based on their feedback, certain redundant items, such as parental ages and occupations, were deleted, and the wording was improved. The questionnaire sought background information (child and parent age, sex, and name) and posed the following questions (Table 1).

**Table 1:** Questionnaire about satisfaction with support.

Questionnaire about satisfaction with support				
Q1) How difficult is it to receive support?				
1, Easy	2, Neither easy nor difficult		3, Difficult	
Q2) To what extent are you satisfied with overall support?				
1, Satisfied	2, Moderately satisfied	3, Neutral	4, Moderately dissatisfied	5, Dissatisfied
Q3) To what extent are you satisfied with:				
・Medical care?		1, Satisfied	2, Neutral	3, Dissatisfied
・Educational support?		1, Satisfied	2, Neutral	3, Dissatisfied
・Administrative support?		1, Satisfied	2, Neutral	3, Dissatisfied
・Familial support?		1, Satisfied	2, Neutral	3, Dissatisfied
Q4) Free-text comments				

Additional items explored why respondents were (dis)satisfied with different types of support and how that support should be offered. All respondents were asked to agree to participate and to self-complete the questionnaire. Questions 1-3 were closed-end questions, and Question 4 was open-ended.

Analysis

First, we compared the Q1 and Q2 responses of parents whose children did and did not exhibit hearing loss. The average hearing levels of both ears were calculated; all 101 children were categorized into those with better hearing (£30 dB; n = 33; normal group) and worse hearing (>30 dB; n = 68; hearing loss group). We performed between-group comparisons of the difficulty experienced when seeking support (Q1) and satisfaction with overall support (Q2).

Next, we compared the responses of those satisfied and unsatisfied with each type of support (Q2 and Q3). For Q2, “(dis)satisfied” and “moderately (dis)satisfied” were classified as (dis)satisfied; for Q3, only “(dis)satisfied” parents were classified as (dis)satisfied. We evaluated differences in the average hearing levels of both ears and current age (months) between the groups. Based on the literature [16], and given the many complaints about regional disparities in the free-text comments, we conducted an additional statistical analysis by residential area. Those who lived in our city, Nagasaki, where two dedicated hearing testing institutions are located, and in two other cities, Ōmura and Isahaya, within a 30-minute drive, were defined as residents of the “suburbs of Nagasaki city” (n = 41), whereas those who lived in other municipalities were defined as residents of the “environs” (n = 60).

Statistical analyses

All statistical analyses were performed using the JMP v16.0 Pro software package (SAS Institute Inc., Cary, NC, USA). Normality was evaluated by drawing distribution plots and histograms. The Shapiro-Wilk test showed that neither average hearing level nor current age was normally distributed; therefore, these comparisons were conducted using the Wilcoxon rank-sum test. Nominal scales (for residential areas) were compared using the Chi-square test or Fisher’s exact test, with the latter applied when p-values differed. The required sample size was calculated. The correlation between satisfaction and familial support was excluded from the analysis because only two respondents were “dissatisfied.” Binomial logistic regression was used to identify significant independent factors that contributed to satisfaction for each type of support. For all analyses, p < 0.05 was considered statistically significant.

Ethics

Ethical approval was obtained from our Institutional Ethics Committee (approval no. 20111627) in November 2020. This study adhered to the principles of the Declaration of Helsinki, and written informed consent was obtained from all participants.

## Results

The first visit to a dedicated hearing test institution occurred between 0 and 328 days after birth (mean: 67.2, standard deviation (SD): 71.4 days). The questionnaires were completed when the children were aged 13-131 months (mean: 70.9, SD: 36.2 months). The questionnaire collection rate for each age group was as follows: 40 cases (39.6%) for children aged one to four years, 44 cases (43.6%) for children aged five to eight years, and 17 cases (16.8%) for children aged nine years and above. The rate was slightly lower for the older age groups but generally similar otherwise. During the study period, the NHS referred 50 cases of bilateral and 51 cases of unilateral ear loss to us. The final hearing level ranged from 7.0 to 105.0 dB (mean: 51.5, SD: 27.7 dB). A definitive diagnosis of hearing loss was based on auditory brainstem or steady-state auditory response combined with auditory behavioral observation, audiometry, and conditioned orientation response. The principal causes of hearing loss were as follows: external and middle ear malformations (n = 11), otitis media with effusion (n = 9), hereditary hearing loss (n = 7), inner ear malformations (n = 7), 21-trisomy (n = 4), congenital cytomegalovirus infection (n = 3), psychomotor retardation (n = 3), enlarged vestibular aqueduct (n = 1), Waardenburg syndrome (n = 1), and unknown (n = 52); no case exhibited progressive hearing loss.

Difficulty in receiving support (total, medical, educational, administrative, and familial support)

**Figure 1:**
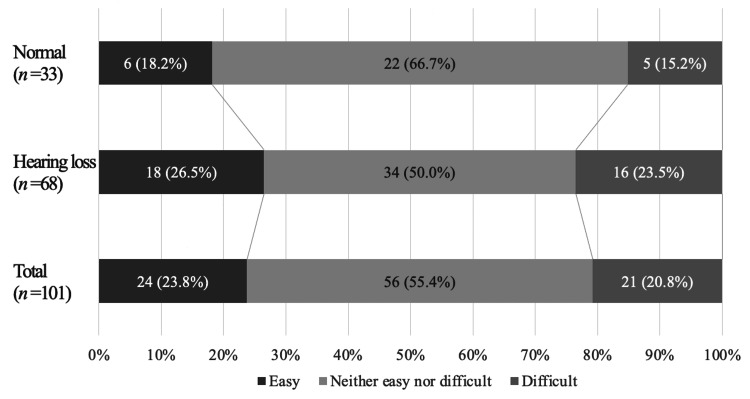
Difficulty encountered when seeking support. The percentage of respondents who answered “Easy” was lowest among parents of children with normal hearing normal group.

For Q1, 24 (23.8%) of respondents replied “Easy,” and the remaining 77 (76.2%) replied “Neither easy nor difficult” (56, or 55.4%) or “Difficult" (21, or 20.8%). Most respondents in both the hearing loss and normal groups answered “Neither easy nor difficult,” but only 18 (26.5%) of the hearing loss group and six (18.2%) of the normal group replied “Easy” (Figure 1).

Satisfaction with overall support

**Figure 2:**
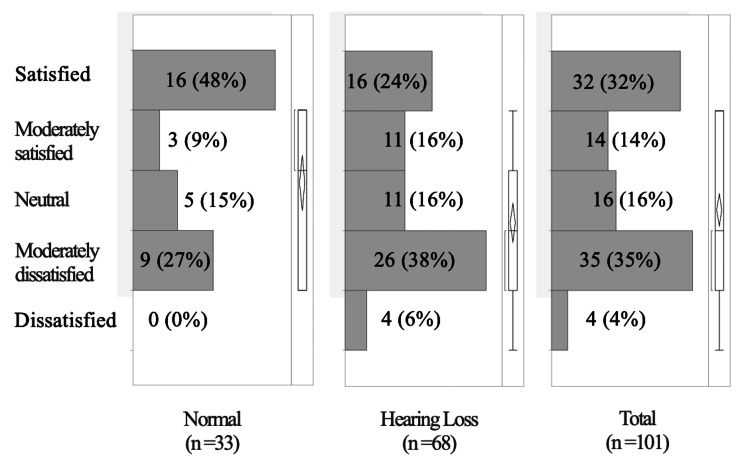
Satisfaction with overall support. The boxplot to the right shows the shortest range; the diamond and bar indicate the mean and median. The bar to the left of the boxplot contains 50% of the data. Despite the two peaks, 27% of parents were “Moderately dissatisfied,” even within the normal group.

For Q2, about half, 16 (48%), of the normal group were “Satisfied,” but 26 (38%) of the hearing loss group were “Moderately dissatisfied”; this was the most common answer. As a result, 35 (35%) were “Moderately dissatisfied” and 32 (32%) were “Satisfied,” such that there were two peaks. Even in the normal group, nine (27%) were “Moderately dissatisfied”; in the hearing loss group, 30 (44%) were “Moderately dissatisfied” or “Dissatisfied” (Figure 2).

Satisfaction with each support

**Figure 3:**
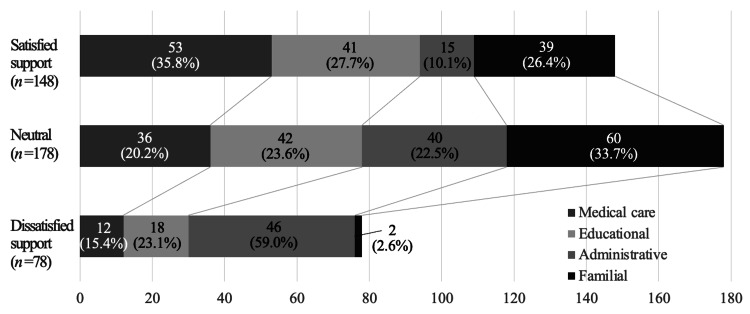
Satisfaction with each type of support. Medical care was associated with the greatest satisfaction (“Satisfied,” n = 53, 35.8%), and administrative support with the worst (“Dissatisfied”, n = 46, 59.0%). The upper and lower figures are absolute and proportional “Satisfied,” “Neutral,” and “Dissatisfied” responses.

Figure 3 presents the responses to Q3 (multiple responses allowed, 404 comments in all), divided into “Satisfied” (148), “Neutral” (178), and “Dissatisfied” (78 answers). In terms of satisfaction, medical support predominated (n = 53, or 35.8%), followed by educational support (n = 41, or 27.7%), familial support (n = 39, or 26.4%), and administrative support (n = 15, or 10.1%). In terms of dissatisfaction, administrative support predominated (n = 46, or 59.0%), followed by educational support (n = 18, or 23.1%) and medical care (n = 12, or 15.4%). In contrast, only two respondents (2.6%) were dissatisfied with familial support.

Analysis results

Statistical correlations between satisfaction with each support (with the exception of familial support, as only two respondents were “Dissatisfied”), and the average hearing level (Table 2) and current age (months) are listed in Table 3. The average hearing level was significantly better among those who were satisfied with the total support than among those who were not (Table 2). The current age was markedly lower among those satisfied with medical care than among those dissatisfied with it (Table 3).

**Table 2:** The statistical analysis results of the correlation between satisfaction and average hearing level. *Statistically significant difference IQR: interquartile range

Support	Answer	Average hearing level (dB) (Median (IQR))	p-value
Total support	Dissatisfied	50.0 (40.0-85.0)	0.0263*
	Satisfied	40.0 (26.3-66.3)	
Medical care support	Dissatisfied	42.1 (33.3-65.6)	0.2944
	Satisfied	55.0 (33.3-65.6)	
Educational support	Dissatisfied	47.5 (29.5-80.5)	0.1340
	Satisfied	61.5 (45.0-93.8)	
Administrative support	Dissatisfied	54.0 (39.0-84.2)	0.6914
	Satisfied	60.0 (40.0-86.5)	

**Table 3:** The statistical analysis results of the correlation between satisfaction and current age in months. *Statistically significant difference IQR: interquartile range

Support	Answer	Current age (months) (Median (IQR))	p-value
Total support	Dissatisfied	79.0 (40.0-101.0)	0.6696
	Satisfied	76.0 (34.5-100.0)	
Medical care support	Dissatisfied	89.5 (68.3-110.5)	0.0324*
	Satisfied	63.0 (31.5-95.0)	
Educational support	Dissatisfied	60.0 (20.8-100.3)	0.1013
	Satisfied	89.0 (63.5-100.0)	
Administrative support	Dissatisfied	66.5 (31.0-100.0)	0.3398
	Satisfied	90.0 (56.0-98.0)	

Table 4 lists the results of the binomial logistic regression identifying variables that contributed to satisfaction with each type of support, except familial support. The average hearing level was negatively correlated with total support satisfaction. Current age was negatively correlated with medical care satisfaction but positively correlated with educational support satisfaction.

**Table 4:** Prognostic variables for satisfaction for each support. *Statistically significant difference

Support	Variable	Adjusted odds ratio	95% CI	p-value
Total support	Average hearing level (dB)	0.219	0.048-0.994	0.0455*
Medical care support		1.016	0.990-1.043	0.2054
Educational support		1.015	0.995-1.039	0.1483
Administrative support		1.004	0.981-1.027	0.7349
Total support	Current age (months)	0.998	0.986-1.010	0.7055
Medical care support		0.981	0.961-0.999	0.0441*
Educational support		1.019	1.001-1.038	0.0377*
Administrative support		1.010	0.993-1.028	0.2069

Table 5 shows the results of a Chi-square test exploring the correlation between residential area and satisfaction. For those living in the suburbs of Nagasaki city, educational satisfaction was significantly higher than that of residents of the environs (χ² = 4.514, p = 0.0336). Although statistical significance was not attained, suburban residence was associated with more administrative support satisfaction than residence in the environs (χ² = 3.459, p = 0.0629).

**Table 5:** Results of the Chi-square test of residential areas and satisfaction. *Statistically significant difference

Residence	χ^2 ^value	p (Prob>|ρ|)
Total support	1.815	0.1779
Medical care support	2.201	0.1380
Educational support	4.514	0.0336*
Administrative support	3.459	0.0629
Family support	0.002	0.9624

Free-text comments

Of the respondents, 39 (38.6%) wrote free-text comments. Most of those who were “Satisfied” stated that the support was well-explained and administered by professional and approachable individuals. Most of those who were “Dissatisfied” with overall support stated that it varied by residential area, was difficult to comprehend, and that their views on support were not consistent with those of the person in charge. In addition, financial (administrative) support was deemed to be slow, and both educational and medical support were considered slow and inadequate.

## Discussion

In this study, satisfaction with total support was lowest among parents of children diagnosed with severe hearing loss after NHS. Among parents of children who wanted support when their children were referred after the NHS, only 23.8% (Figure 1) responded that support was “Easy.” In their free comments, many requested information on support, even if their children had only slight to mild hearing loss. These families did not know where to turn.

Khairi et al. [17] reported that a considerable proportion of mothers whose infants received false-positive NHS results experienced unacceptable anxiety and required help, which was provided. Fitzpatrick et al. [18] found that parents of young children with hearing loss, including mild bilateral or unilateral hearing loss, preferred speech and language development to amplification. In the present study, 27% of respondents in the normal group were moderately dissatisfied; only 18% found it “Easy” to obtain support (Figure 1). Our findings are consistent with those of previous studies [17] and emphasize that support is crucial, even when children have slight to mild hearing loss.

Of the various types of support, satisfaction with administrative support was the lowest (Figure 3), financial help was lacking, and regional disparities were apparent. Poorer reading proficiency in children was associated with lower economic status [19]. It is possible that satisfaction with medical care was lower among parents with older children, who thus underwent earlier NHS (Tables 3-4). Since the quality of medical care has been improving year by year, it may be inferred that, as a result, parents of more recently born children are more satisfied with their medical support. Notably, although statistical significance was lacking, the children of parents dissatisfied with educational support tended to have better hearing levels than those of parents who were satisfied. Also, although statistical significance was lacking, the children of parents dissatisfied with educational support tended to be younger than those of satisfied parents. Satisfaction with educational support may gradually increase with the child’s age. These observations support the results of a previous report [20]: educational placement changed as children gained experience with cochlear implants and classroom emphasis on speech and auditory skills increased in special schools for those with hearing impairments. Indeed, one free-text comment asserted that some regular schools do not offer support to children with mild hearing loss. Hearing aid assistance systems are available to students at schools for the deaf, but not at some regular schools; this created parental dissatisfaction. In terms of residential area, satisfaction with educational support was markedly lower among parents living in the environs (Table 5), for whom daily school trips were long. Familial support was less dependent on the place of residence, and medical institutions were infrequently visited. Bush et al. [16] reported that educational, distance, accessibility, and socioeconomic factors can constitute barriers to the timely diagnosis and treatment of congenital hearing loss in children residing in rural areas. Regional differences in NHS early intervention services have been reported [21]; children with hearing loss living in remote areas have suffered delays in early diagnosis and intervention [22,23]. To eliminate regional disparities, it is essential to create well-staffed satellite facilities that better disseminate support. Adequate funding and trained personnel are essential.

The present study has some limitations as follows. First, the correlation between satisfaction and family support could not be evaluated because there were only two “Dissatisfied” responses. This gap may affect the reported satisfaction with each type of support and the results of the statistical analysis, and it is necessary to increase the sample size. Second, only 13 children had unilateral hearing loss at the end, which was not a sufficient number of cases to perform statistical analysis. Therefore, children with unilateral hearing loss were included in the “hearing loss group” for analysis. Larger sample sizes are required in future investigations of the utility of various types of support, including the specific content and the locations that offer parental consultations and rehabilitate children with hearing impairments. We also did not explore whether children had disabilities other than hearing loss; such children would require greater support if their parents were to be “Satisfied.”

On the other hand, a strength of this study is that it evaluates the specific content and parental satisfaction with the support provided to parents of children diagnosed with hearing loss by the NHS, something that has rarely been evaluated to date.

## Conclusions

In conclusion, parents do not know how to obtain support or what support is available. Parental satisfaction was particularly low when children experienced hearing loss, and even when children had normal hearing. Satisfaction with administrative support was low, while satisfaction with educational support was high, but only among suburban residents. To address these issues, it is imperative to clarify what support is available, where to find it, and eliminate regional disparities in support policies and education. Additionally, government spending should be increased, and support workers should be trained. We hope that our findings will assist the parents of children referred by the NHS in the future.
